# Motor ability development by integrating small-sided games into physical education class

**DOI:** 10.3389/fpsyg.2024.1259924

**Published:** 2024-01-22

**Authors:** Quan Zhi Li, Qun Fang, Xi Tang Zhao, Wan Peng

**Affiliations:** ^1^School of Sport and Health, Nanjing Sport Institute, Nanjing, Jiangsu, China; ^2^School of Physical Education, Qingdao University, Qingdao, Shandong, China

**Keywords:** small-sided game, physical education, fitness, motor ability, team ball sports

## Introduction

Approximately 70% of children and adolescents worldwide fail to meet the recommended level of physical activity (He et al., 2021). As obesity-related health issues become increasingly serious, researchers focus on the essential role of physical education (PE) in health promotion for children and adolescents (Hills et al., 2015). PE incorporates a variety of lifetime activities into curricula. School-based interventions are considered an applicable and effective strategy to increase daily PA for children and adolescents, thus leading to a healthy, active lifestyle in adulthood (Bukowsky et al., 2014). Abundant evidence has shown positive effects of PE on physical, affective, social, and cognitive promotion (Bailey, 2006; Donnelly et al., 2016). A recent project provided evidence for the positive influence of a PE program on motor and cognitive development for pre-school children (Battaglia et al., 2020). After 16 weeks of PE classes, significant improvement was identified in locomotor and object control skills. The children also indicated better pre-literacy skills, implying academic success in the future.

Comprehensive motor development in endurance, strength, power, balance, and flexibility is of great importance to children and adolescents (Lubans et al., 2010; van Baak et al., 2021). However, the current PE places a specific attention to quantitative aspects of PA and health-related components of physical fitness such as aerobic fitness, muscular endurance, flexibility, and body composition, but a lack of emphasis on skill-related components in agility, coordination, power, speed, and balance (Myer et al., 2015; Cho et al., 2022; Hao and Yang, 2022; Hastie et al., 2022). Research has shown that PE classes often fail to provide sufficient exercise intensity to induce changes in body tissue composition and physical fitness (Domaradzki et al., 2020). Class activities that incorporate short-term, high-intensity interval training (HIIT) protocols are considered a promising approach to adequate exercise intensity. In addition to the concern with intensity, quality PE requires the class beyond “effective” (Ennis, 2017). By taking advantage of knowledge and theories across disciplinaries, researchers look for novel pedagogical strategies to enhance motivation and facilitate learning (Fang et al., 2022; Pang et al., 2023).

Recently, small-sided games (SSG) have raised a wide interest among PE teachers due to practical considerations. The main reason for the increasing notice on SSG can be attributed to the limited campus space which is particularly evident in Asian countries (Fang et al., 2023). For example, a typical class size of elementary and middle schools in China is about 40. A soccer game in a full field usually accommodates approximate 20 students to play at the same time, leaving the other half of the class not engaged in the game play. Additionally, the large ratio of student to teacher (40:1) also raises a challenge for PE teachers to tack care of the whole class when the students perform individual practice. In this sense, SSG can help PE teachers to organize and manage the class in an efficient manner. A teaching environment requires creative ways to keep appropriate class size in PE (Ennis, 2017). Therefore, the current study aims to lay a theoretical foundation for a wide application of SSG under PE settings by critical analysis on the characteristics and effects of the training modality.

## Features of SSG in support of application to PE

As an effective, motivating, and fun exercise that stimulates physiological responses and neuromuscular adjustments, SSG has been widely applied in team ball sports (Ouertatani et al., 2022). The effectiveness of SSG leads to a reasonable implication of applying this PA modality to enhance teaching and learning in PE settings.

Flexible design is a prominent factor to justify successful application of SSG to PE class. Instructors can easily modulate SSG format by the number of players (Moreira et al., 2016), pitch configuration (Chaouachi et al., 2014; Stevens et al., 2016), time, and rules (Clemente et al., 2021a,b) to achieve diverse objectives. Typically, altering the number of players and pitch size is the common approaches to organizing the training modality in practice, which produces significant influences on training intensity (Clemente et al., 2021a,b). Research has shown a positive relationship between relative area and intensity. Reducing the number of players in a constant pitch size increases the relative area for individual players, which stimulates a higher physical load (Moreira et al., 2016).

It is also important to notice the advantage of SSG in replicating the movement patterns of competition (Giménez et al., 2020). This enables players to develop sport-specific fitness and skills required in a high-intensity game situation. Researchers compared soccer players taking 3 vs. 3 SSG with their counterparts performing interval running in an 8-week training program (Radziminski et al., 2013). Changes in VO_2max_ and soccer-specific skills are greater in the SSG group than the interval running group. The prominent improvements are attributed to the increased number of accelerations completed in SSGs compared with the full-size football field (Giménez et al., 2020; Castillo Alvira et al., 2021). Similarly, a systematic review on SSG in volleyball indicated that skill-based conditioning training, which was carried out in the formats of 5 vs. 5, 5 vs. 4, and 5 vs. 3, successfully simulated the high-intensity physiological demand of national-level competition and improved physical fitness in speed, vertical jump, spike jump, agility, upper-body muscular power, and maximal aerobic power (de Oliveira Castro et al., 2021).

High intensity makes SSG a time-efficient training modality for PE (Arslan et al., 2020; de Oliveira Castro et al., 2021; Stojanović et al., 2021). In a typical 45 minutes of PE class, the time for physical activity can be limited excluding the required activities such as warm-up, instructions, and cool down. An alternative to warm-up routines contain a series of high-intensity, short-duration activities inducing a post-activation potentiation effect (Zois et al., 2015). Instructors may consider using SSG as specific conditioning training, which enhances physiological demands in practice (Davids et al., 2013; Mazurek et al., 2018; Arslan et al., 2020; Jurišić et al., 2021).

Due to the intense and intermittent activities in SSG, research evidence has shown advantages of SSG over HIIT. While SSG induces comparable effects on physical performance to HIIT protocols, additional benefit in technical skills has been reported in basketball (Delextrat and Martinez, 2014; Arslan et al., 2022) and soccer (Chaouachi et al., 2014; Arslan et al., 2020). In a meta-analysis on effects of soccer SSG compared with conventional endurance training, no significant difference was identified in endurance performance between the two training groups (Moran et al., 2019). Researchers recommended SSG as a more efficient protocols for simultaneous development of endurance and skills. Despite the benefits of SSG, it is worth noting specific advantages of HIIT in promoting physiological abilities, including maximal oxygen uptake, aerobic performance, linear sprint ability, and repeated sprint ability (Clemente et al., 2021a,b). Researchers and practitioners thus attempt to combine SSG with HIIT for better effects on running performance (Clemente and Sarmento, 2021). Compared with SSG-only approach, the combined protocol increased the acute mechanical load and high-intense running stimuli (Clemente and Sarmento, 2021). Enhanced physical performance in linear sprint, repeated sprint, agility, and countermovement jump have been reported in the combined SSG for young soccer players (Arslan et al., 2021).

Another feature in support of applying SSG to PE is the greater enjoyment than traditional practice. In a study involving youth soccer players, SSG induced greater enjoyment than interval training (Los Arcos et al., 2015). Physical education teachers could use this during specific soccer sessions to maintain a high level of motivation among the students, which induces engagement in class activities (Araújo et al., 2016; Larsen et al., 2018; Sahli et al., 2020). Motivational exercises in PE classes can stimulate physical effort, personal feeling, and enjoyment (Aydi et al., 2023). SSG induces higher physiological responses while maintaining players' motivation. Therefore, SSG increases the amount of time spent in playful and enjoyable activities by allowing learners to experience simulations of competitive team games.

The above analysis identified key features of SSGs to facilitate its application to PE classes, including flexible design, replication of game patterns, high intensity, and enjoyment. By modulating pitch size, player numbers, rules, and play time, instructors can implement the SSG-based class in a time-efficient manner, which effectively enhances strength conditioning and sport-specific skills of the students.

## PE class design based on team ball SSG

While the features of SSG imply promising applications to PE class, in this section, further investigations focus on organizations of SSG in team ball sports. Soccer is the mostly studied team ball sport in the existing literature. Organizations of SSG in soccer can be manipulated by variations in pitch area, player number, and rules (Hill-Haas et al., 2011; Halouani et al., 2017). In addition, experienced coaches are skilled at making an adaptive session by altering game rules. A commonly applied approach is to limit the number of touches. Compared with free touch, demanding a maximum of 2–3 touches can increase mental workload as well as intensity during game play (Hill-Haas et al., 2010). Another way to modify game rules is the neutral player. The neutral player transitions to the team in possession to create unbalanced situations between defensive and offensive teams (Mallo and Navarro, 2008). Scoring rules are also determinants of performance in SSG. Researchers have found that the size of goals and presence of goalkeepers influence players' behaviors. In general, the presence of goalkeepers stimulates players' motivation and effort in both attack and defend, which increased the physical load (Dellal et al., 2008).

SSG in basketball is often organized in 2-, 3-, and 4-a-side in half court (Klusemann et al., 2012; Zeng et al., 2021). For example, in a 6-week intervention program, participants underwent 2 vs 2 training in a size of 28m × 7.5m, which induced greater improvement in defensive agility, shooting skills, and upper body power than HIIT (Delextrat and Martinez, 2014). Rule modifications are also applied to organize basketball SSG. In a series of 3 vs. 3 basketball games, specific rules were given to the attacking team, including seven seconds possession and three passes maximum per attack (Camacho et al., 2021). The constraints in time and pass placed a higher demand on cognitive and skill performance, which provide a good example of organizing basketball SSG by rule modifications. Another factor to impose a high level of intensity is the work to rest ratio. In a half court 3 vs. 3 basketball training, researchers designed long-intermittent SSGs which consisted of three 4-min bouts interspersed by 2-min passive recovery, and short-intermittent SSGs which consisted of six 2-min bouts interspersed by 1-min passive recovery (Sansone et al., 2020). The work to rest ratio is a key factor in designing basketball SSG in that shorter regime induces higher technical demands.

SSG in volleyball can be implemented by modified court size. Pekas et al. (2019) conducted 2 vs. 2 in a size of 7m × 3m and 3 vs. 3 in 12m × 6m for young volleyball players. The SSG protocol induced greater improvement in lower body explosive power (i.e., block jump, spike jump, and countermovement jump) than controls. It is interesting to find SSG an effective teaching approach for volleyball novices. Researchers designed 2-a-side in four court configurations which resulted in the area/player ratios of 4.5 m^2^ (3.0 m x 3.0 m), 8.0 m^2^ (4.0 m x 4.0 m), 10.58 m^2^ (4.6 m x 4.6 m), and 13.52 m^2^ (5.2 m x 5.2 m). In addition to the benefits in technical skills, tactical behaviors indicated significant improvement after the 3-day training. School-based programs have shown the feasibility of implementing SSG in volleyball PE classes. In an 8-month after-school volleyball program for high school students, significant effects were identified in promoting physical fitness and reducing aggressive behaviors (Trajković et al., 2020).

## Conclusion

Evidence-based literature indicated SSG an effective access to high-intensity exercise, suggesting a feasible application of this training approach to PE classes. SSG is characterized by flexible design, replication of game patterns, high intensity, and enjoyment. Integrating SSG into PE classes can effectively stimulate engagement and moderate-to-vigorous physical activities. Based on the characteristics of SSG, PE class design was discussed on soccer, basketball, and volleyball. By modifying pitch size, player numbers, rules, and work to rest ratios, PE teachers can effectively engage students in SSG which promotes physical fitness in concurrent with motor skill learning.

## Author contributions

QL: Data curation, Investigation, Writing—original draft. QF: Methodology, Writing—review and editing. XZ: Formal analysis, Methodology, Writing—review and editing. WP: Formal analysis, Supervision, Writing—review and editing.
